# A molecular pathology method for sequential fluorescence in situ hybridization for multi-gene analysis at the single-cell level

**DOI:** 10.18632/oncotarget.10245

**Published:** 2016-06-23

**Authors:** Linping Hu, Xiuxiu Yin, Jiangman Sun, Anders Zetterberg, Weimin Miao, Tao Cheng

**Affiliations:** ^1^ State Key Laboratory of Experimental Hematology, Institute of Hematology, Blood Disease Hospital, Chinese Academy of Medical Sciences and Peking Union Medical College, Tianjin China; ^2^ The Department of Oncology-Pathology, Karolinska Cancer Institute, Karolinska Institute, Stockholm, Sweden; ^3^ Union Stem Cell and Gene Engineering Co. Ltd, Tianjin China

**Keywords:** molecular pathology, genotyping, sequential FISH, single cell, multi-gene detection

## Abstract

Multi-gene detection at the single-cell level is desirable to enable more precise genotyping of heterogeneous hematology and oncology samples. This study aimed to establish a single-cell multi-gene fluorescence *in situ* hybridization (FISH) method for use in molecular pathology analyses. Five fluorochromes were used to label different FISH gene probes, and 5 genes were detected using a five-color FISH protocol. After the first hybridization, the previous FISH probe set was stripped, and a second set of five-color FISH probes was used for rehybridization. After each hybridization, the fluorescence signals were recorded in 6 fluorescence filter channels that included DAPI, Spectrum Green^™^, Cy3^™^ v1, Texas Red, Cy5, and PF-415. A digital automatic relocation procedure was used to ensure that exactly the same microscopic field was studied in each stripping and hybridization cycle. By using this sequential stripping and rehybridization strategy, up to 20 genes can be detected within a single nucleus. In conclusion, a practical molecular pathology method was developed for analyzing multiple genes at the single-cell level.

## INTRODUCTION

Fluorescence *in situ* hybridization (FISH) is a popular technique that uses fluorochrome-labeled DNA probes to detect the deletion, amplification and translocation of one or several DNA molecules within chromosomes. FISH combines cutting-edge molecular biology techniques with an advanced computerized microscope system. It can detect genetic abnormalities at the visible single-cell level, thereby avoiding false results from pooled samples of mixed cells [1, 2].

While FISH is useful for detecting certain chromosomal abnormalities [1, 3, 4], the number of genes that can be simultaneously detected is limited. Currently, most FISH kits that are used in the clinic detect one or two genes and involve the use of one or two fluorescence colors at a time. The use of quantitative multi-gene FISH (qmFISH) has increased in recent years[5–8]; however, the number of detected fluorochromes and genes is usually less than 5 due to the capacity of currently available fluorescence filter sets. To detect more genes in a sample, we have successfully developed a sequential stripping and rehybridization strategy that increases the number of detectable genes to up to 20 in a single cell. This method can be used for more precise molecular subtyping[9] or for clonal evolution [10–12] studies of various types of diseases.

## RESULTS

### Preparation of FISH probes

**Figure 1:**
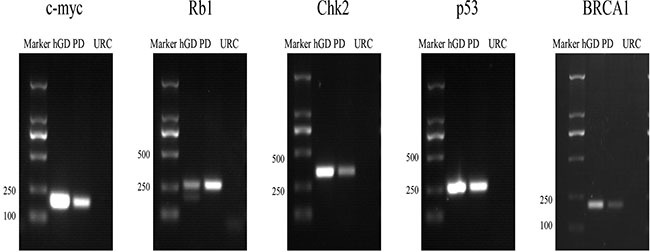
PCR detection of the target genes contained in each BAC clone, such as c-myc, Rb1, Chk2, p53 and BRCA1 Marker: DNA molecular standard; hGD: human genome DNA, used as a positive control; PD: FISH probe DNA; URC: unrelated BAC clone DNA, used as a negative control.

**Figure 2:**
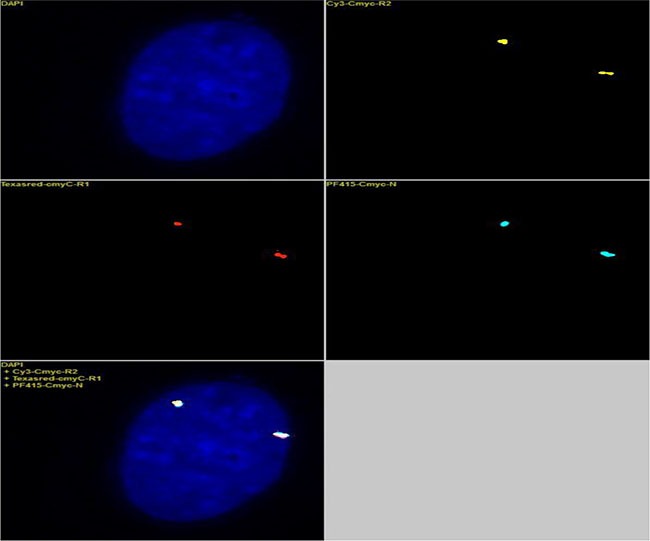
The 3 BAC clones (Table 1) that covered the c-myc gene locus were labeled with yellow, red and blue fluorochromes and hybridized with normal diploid fibroblasts There were two fluorescent signal points for each yellow, red and blue color, suggesting that each BAC clone detected two copies of the locus. The results further showed that the yellow, red and blue probes bound to the same genetic locus, suggesting that the three BACs targeting the c-myc locus could be used either independently or together as a c-myc FISH probe.

As more disease genes have been discovered in recent years, there is an urgent need for multi-gene parameter assays that enable more precise molecular pathological evaluations. Because not all FISH probes for disease genes are commercially available, we have developed the FISH probes used in this work ourselves. Briefly, bacterial artificial chromosome (BAC) clones were purchased from Invitrogen and then confirmed by PCR detection of the target genes or STSs (Figure 1). Because fluorescence signal intensities are largely correlated with the length of the genomic DNA fragment targeted by the probe, we typically used a genomic contig containing 2–3 BACs to prepare a FISH probe that yielded a satisfactory signal intensity. For example, a three-BAC genomic contig targeting the c-myc gene was selected from the UCSC genome map Table 1. After the BAC clones were confirmed by PCR, the utility of each BAC clone as part of a FISH probe was further determined based on the results of preliminary FISH experiments (Figure 2). All three c-myc BAC clones detected specific signals with low levels of non-specific background noise (Figure 2), which suggested that the tested BACs do not contain highly repetitive sequences. Furthermore, the fluorescence signals detected by all three BAC clones of the c-myc contig overlapped, confirming that they all detected the same genomic locus (Figure 2).

**Table 1:** c-Myc gene probe labeling

BAC clone	Fluorochrome
RP11-367L7	PF590-dUTP (Red)
RP11-944J14	PF555-dUTP (Yellow)
RP11-440N18	PF415-dUTP (Blue)

### Test of qmFISH in normal diploid cells and various types of diseased cells

**Figure 3:**
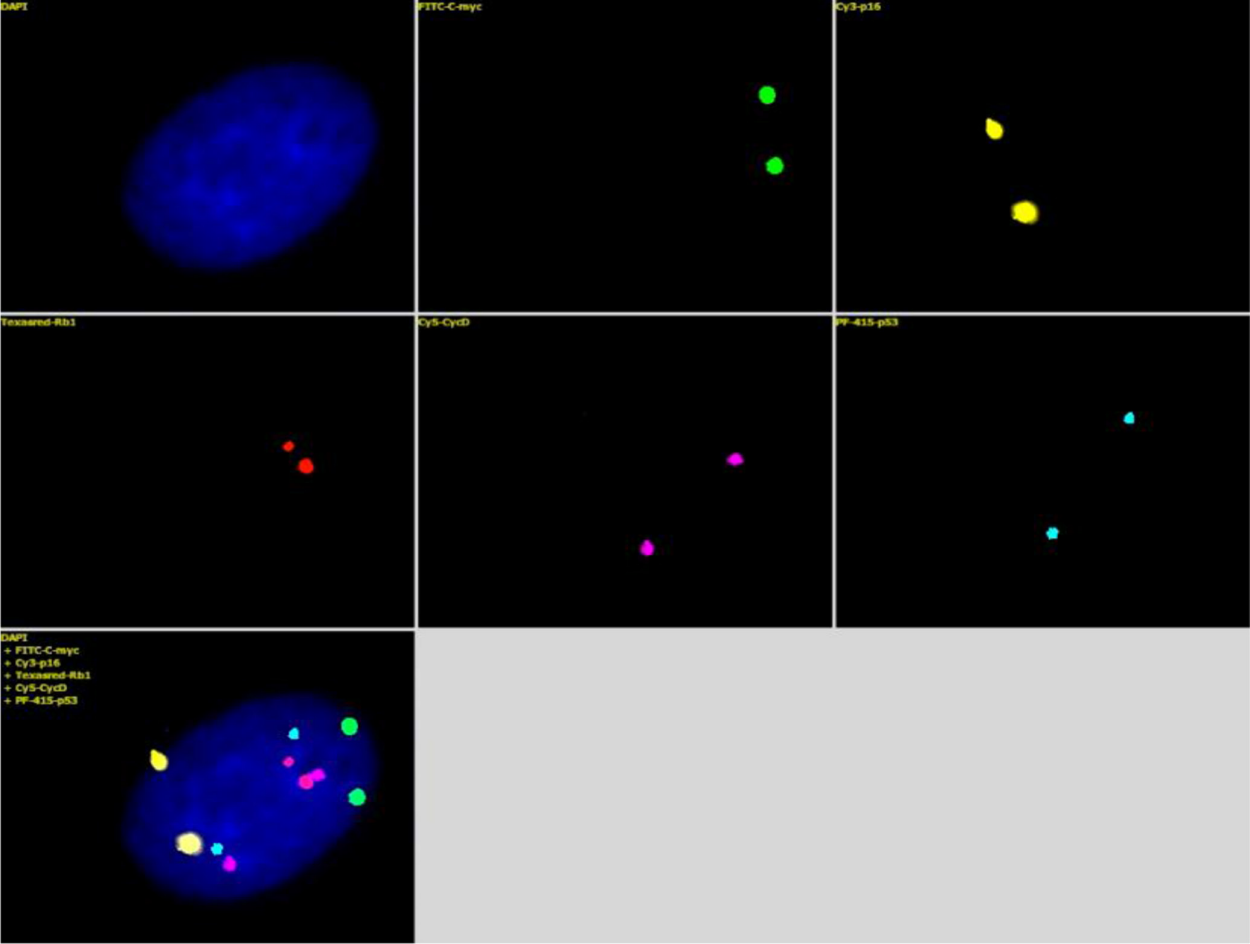
The mixture of the five FISH probes, Green-c-myc (green), PF555-P16 (yellow), PF590-Rb1 (red), HyPer5-CycD (purple), and PF415-p53 (blue) (Table 2), was hybridized with the human diploid fibroblasts Each probe detected two clear fluorescence signals within a single nucleus, and the five probes displayed 10 total fluorescence signals. The fluorescence image was clear with a high signal/noise ratio.

**Figure 4:**
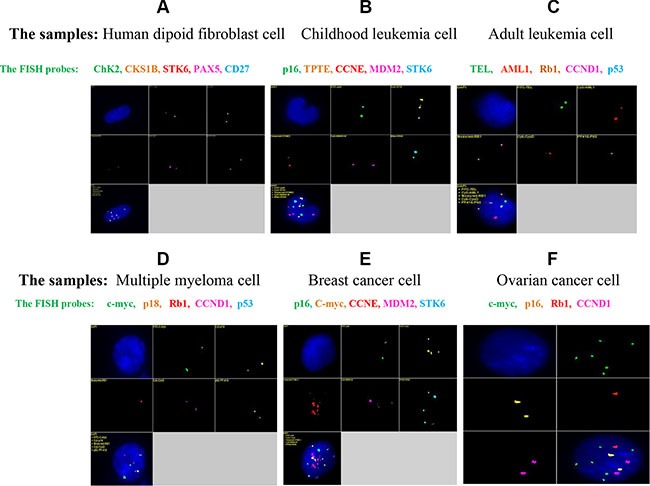
qmFISH was tested on samples of normal diploid cells (A) as well as cell samples from childhood leukemia (B), adult leukemia (C), multiple myeloma (D), breast cancer (E) and ovarian cancer (F) Multi-color probe sets were designed for each disease type and were first tested on human diploid fibroblasts before being applied to the disease samples. Each probe detected two copies of the fluorescence signal in the normal diploid cells (A), and some probes identified interesting genetic abnormalities in the diseased cells (B, C, D, E and F). Specifically, there were 3 copies of the TPTE gene in the childhood leukemia sample (B); one copy each of the CCND1 and p53 genes was deleted in the adult leukemia sample (C); one copy of Rb1 was deleted in the multiple myeloma sample (D); c-myc, CCNE, MDM2 and STK6 all displayed marked amplification that presented as at least 4 copies in the breast cancer sample (E); and c-myc was significantly amplified (6 copies) in the ovarian cancer sample (F).

A five-color probe set that included Green-c-myc (green), PF555-P16 (yellow), PF590-Rb1 (red), HyPer5-CycD (purple), and PF415-P53 (blue) was generated by nick translation labeling (Table 5). The probe set was tested on a normal diploid fibroblast sample; each probe detected two copies of a fluorescent signal within a single nucleus, and the five-probe set detected 10 total signals. The intensities of all of the fluorescent signals were satisfactory, and the images were clear with little non-specific background signal (Figure 3). Then, diverse probe sets (for different disease settings) were generated and tested in different diseased cells (Figure 4B–4F). The quality of the fluorescence signals from the different probes was satisfactory; the images were clear, and the results were unambiguous. Unlike in normal diploid fibroblasts (Figure 4A), some probes detected interesting genetic abnormalities, such as gene amplifications and deletions, in the diseased cells (Figure 4B–4F).

**Table 2:** A five-colour FISH probe set

Gene probe	Fluorochromes
c-myc	Green-dUTP (Green)
P16	PF555-dUTP (Yellow)
Rb1	PF590-dUTP (Red)
CycD	HyPer5-dUTP (Purple)
p53	PF415-dUTP (Blue)

### Development of a sequential qmFISH protocol for multi-gene detection at the single-cell level

**Figure 5:**
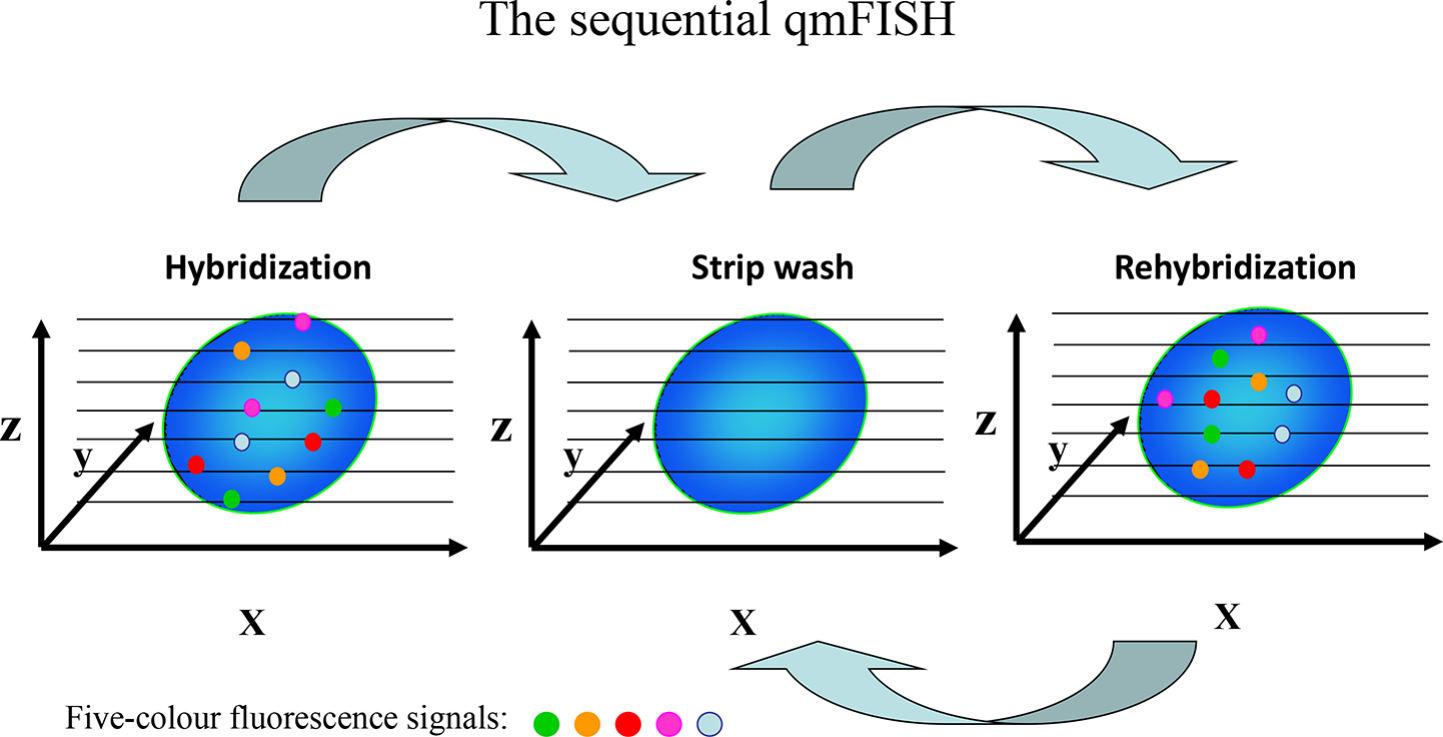
Schematic of the sequential qmFISH strategy After the first hybridization, the slides were strip-washed and then hybridized with a new set of FISH probes. After the rehybridization step, the microscopic field was relocated using a digital automatic procedure. Through several cycles of stripping and rehybridization, multiple genes could be detected within a single nucleus.

**Figure 6:**
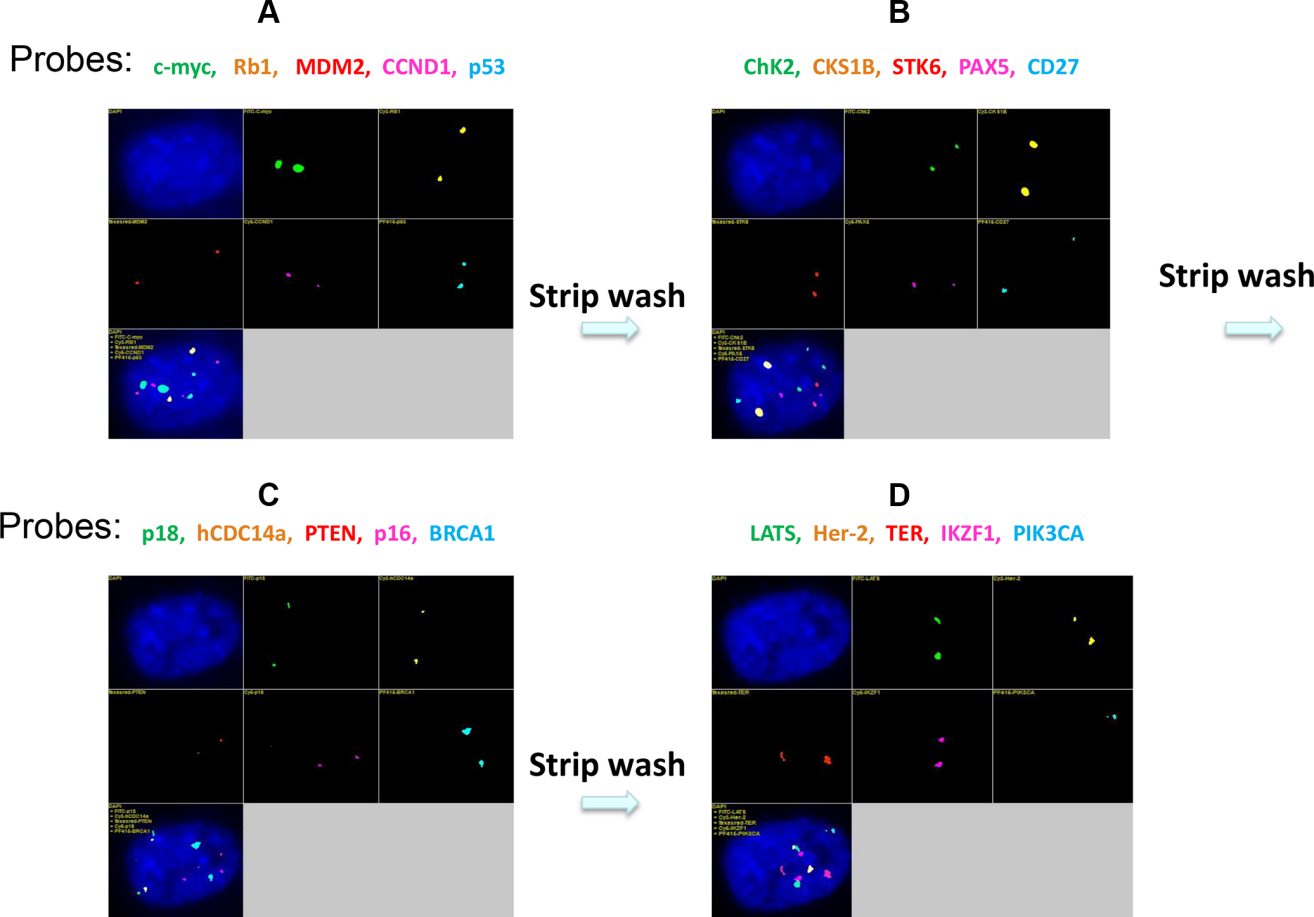
Four sets of 5-color FISH probes were used in this study (Table 3) The images were obtained after hybridization with normal human diploid fibroblasts. After hybridization with the first probe set, a suitable microscopic field was selected in the DAPI channel, and the multi-color fluorescence signals were recorded in the five specific filter channels (**A**). Then, the slides were strip-washed to remove the first set of probes and hybridized with the second probe set (**B**). After rehybridization, the slides were placed under a microscope. The previously selected microscopic field was relocated in the DAPI channel using a digital automatic relocation procedure, and multi-color fluorescence signals were recorded as before. Three cycles of sequential stripping and rehybridization (Figure 6B–6D) enabled the analysis of 20 genes at the single-cell level in the same sample.

To increase the number of genes that can be detected by qmFISH, we tested a sequential qmFISH (stripping and rehybridization) strategy (Figure 5). Briefly, a total of 20 FISH probes were labeled with the designated fluorochromes to make four five-color probe sets (Table 3). In Figure 6, panel a shows a fluorescence image after hybridization with the first probe set, whereas the images in panels b, c, and d represent fluorescence images from the rehybridization steps with the second, third and fourth probe sets, respectively. All 4 images (Figure 6A–6D) were clear, and the fluorescent signals representing the 20 different genes were intense and easily observed. The results suggest that this methodology indeed works well.

**Table 3:** The 20 genes targeted with four sets of five-colour FISH probes

Fluorochrome	Probe set 1	Probe set 2	Probe set 3	Probe set 4
Green-dUTP (Green)	c-myc	Chk2	p18	LATS
PF555-dUTP (Yellow)	Rb1	CKS1B	hCDC14a	Her-2
PF590-dUTP (Red)	MDM2	STK6	PTEN	TER
HyPer5-dUTP (Purple)	CCND1	PAX5	p16	IKZF1
PF415-dUTP (Blue)	p53	CD27	BRCA1	PIK3CA

In conclusion, we have established a high-resolution multi-gene FISH analysis method. By employing a sequential qmFISH strategy, up to 20 genes can be detected within a single nucleus.

## DISCUSSION

Currently, samples from tumor tissues that are collected for gene analysis are not pure; they contain a mixture of tumor cells and surrounding normal cells. Furthermore, tumor cell populations are heterogeneous in that they contain different clones, including cancer stem cells (CSCs) [13, 14]. Therefore, results from these detection assays actually represent an average level of gene expression or amplification. When the percentage of the target cells, such as CSCs [15], is low in the tissue sample, the critical driver mutations or gene expression abnormalities may be missed due to the high level of noise from the background cells. Hence, developing technology for performing single cell gene analysis is highly desired to address these problems.

Thus far, several single cell isolation methods, such as cell cytometry [14] and microfluidic technologies [16], have been developed. Isolated cells can also be used for downstream gene analysis, such as PCR, whole genome/exon sequencing, and RNA-Seq. However, these single cell methods remain in the development stage because of their high cost and relative complexity considering a routine clinical setting. Among all of the current molecular assays, FISH represents one of the few, if not the only, techniques in which genetic abnormalities can be analyzed visually at the single-cell level. However, the number of genes (usually less than 5) that can be detected by FISH is limited.

In this work, we have successfully developed a practical method that allows for the detection of up to 20 genes within a single cell of pathological interest. This was achieved through a combined sequential stripping and rehybridization strategy, a digital microscope field relocation procedure and a high-resolution fluorescence signal acquisition system.

With respect to the sequential stripping and rehybridization protocol, to avoid carryover fluorescence signals after each stripping, the slides were analyzed in each filter channel of the fluorescence microscope to ensure the complete removal of the previous fluorescence signals. A second stripping was sometimes necessary to completely remove residual fluorescence signals before the next rehybridization step. Initially, we were concerned about the durability of the tissues and cells during the multiple FISH procedural cycles, but the stripping and rehybridization procedures were sufficiently gentle and thus did not significantly harm the samples on the slides. The samples can tolerate at least 3 stripping and rehybridization cycles. Hence, up to 20 genes can be detected within a single cell. Therefore, the method is reliable and practical, which is especially useful when a limited number of cells are available from biopsy samples.

## MATERIALS AND METHODS

### Source of patient samples

The hematological and cancer samples were collected from the Blood Disease Hospital Chinese Academy of Medical Sciences and the Cancer Hospital Tianjin Medical University. The FISH experiments with patient samples were approved by the Institutional Ethical Committee of the Institute of Hematology Chinese Academy of Medical Sciences and the Cancer Institute Tianjin Medical University.

### Preparation of FISH probes

BAC clones containing the target genes were selected from the UCSC Genome Bioinformatics Library and confirmed by PCR. Briefly, the BAC DNAs were prepared using a Qiagen plasmid extraction kit and digested with EcoRI before labeling with fluorochrome-linked dUTP (Table 4) by nick translation.

**Table 4:** The fluorochrome-labeled dUTPs used in this study

Fluorochrome	color	Absorbance λ (nm)	Emission λ (nm)
Green dUTP (Green)	Green	496	520
PromoFluor-555-aadUTP (PF555)	Yellow	557	574
PromoFluor-590-aadUTP (PF590)	Red	581	598
PromoFluor-415-aadUTP (PF415)	Blue	418	467
HyPer5 dCTP (HyPer5)	Purple	665	682

### Hybridization

Slides containing tissue or cells were pretreated, denatured with the FISH probes at 85°C for 5 min and then hybridized at 47°C overnight.

### Image acquisition and analysis

After hybridization, the slides were washed twice with SSPE, dehydrated in a gradient of concentrated ethanol solutions, stained with DAPI and examined under a fluorescence microscope (Zeiss Axio Imager Z2) with the following fluorescence filter sets: DAPI (SP-100), Spectrum Green^™^ (MF101), Cy3^™^ v1 (SP-102), Texas Red (SP-107), Cy5 (50) and PF-415 (45). Advanced 3D imaging software (AxioVision Rel. 4.8) was used with the microscope. Specifically, a microscopic field with a monolayer of tissues or cells of interest was selected and located in the DAPI filter channel using automatic relocation software (AxioVision Rel. 4 Module Mark & Find 2). The fluorescence images were recorded using a high-resolution charge-coupled device (CCD) in five filter channels, including Spectrum Green^™^, Cy3^™^ v1, Texas Red, Cy5 and PF-415. The 25–30 layers of stereoscopic tomography images for each photographed cell nucleus were acquired using the Z-Stack module, and then the multiple layers of fluorescence signals were ultimately merged into a single layer to achieve a clear, stable and satisfactory five-color image.

### Sequential quantitative multi-gene fluorescence *in situ* hybridizations (Sequential qmFISH)

After the first hybridization and image recording step, the slides were temporarily stored at 4°C. The probe stripping was performed as follows. The coverslip was removed, and the slides were washed in 2X SSC, placed in 70% formamide at 70°C for 3 min and then quickly soaked in −20°C pre-chilled ethanol. The slides were then hybridized with a new FISH probe set using the same protocol as before. After the rehybridization step, the previous microscopic field was relocated using the automatic relocation software (AxioVision Rel. 4 Module Mark & Find 2). Again, the fluorescence images were recorded with a high-resolution CCD in six fluorescence filter channels: DAPI, Spectrum Green^™^, Cy3^™^ v1, Texas Red, Cy5 and PF-415. By using 3 cycles of stripping and rehybridization, up to 20 genes could be detected within a single cell of pathological interest.

### Evaluation of the results

The specific fluorescence signals should be located within the nucleus, and diverse fluorescence colors should be used to represent the different detected genes. The number and location of the signals should correlate with the normal or abnormal status of the examined genes. In a normal diploid cell, each gene corresponds with 2 copies of fluorescent signal. A gene deletion therefore manifests as the disappearance of 1 or 2 copies of the signal, gene amplification produces multiple copies of the fluorescence signal, and gene recombination creates a fusion of two different fluorescent signals.

## Abbreviations

FISH: fluorescence *in situ* hybridization
qmFISH: quantitative muti-gene FISH
BAC: Bacterial artificial chromosome
CSCs: Cancer stem cells
CCD: Charge-Coupled Device.
